# GeneChip analysis of resistant *Mycobacterium tuberculosis* with previously treated tuberculosis in Changchun

**DOI:** 10.1186/s12879-018-3131-8

**Published:** 2018-05-22

**Authors:** Ming-Jin Zhang, Wen-Zhi Ren, Xue-Juan Sun, Yang Liu, Ke-Wei Liu, Zhong-Hao Ji, Wei Gao, Bao Yuan

**Affiliations:** 1 Department of Laboratory Animals, College of Animal Sciences, Jilin University, Changchun, 130062 Jilin China; 2 Department of Infectious Diseases, Changchun Infectious Hospital, Changchun, 130123 Jilin China

**Keywords:** *Mycobacterium tuberculosis*, Drug resistance, Gene chip

## Abstract

**Background:**

With the widespread use of rifampicin and isoniazid, bacterial resistance has become a growing problem. Additionally, the lack of relevant baseline information for the frequency of drug-resistant tuberculosis (TB) gene mutations is a critical issue, and the incidence of this infection in the city of Changchun has not investigated to date. However, compared with the slow traditional methods of drug susceptibility testing, recently developed detection methods, such as rifampicin and isoniazid resistance-related gene chip techniques, allow for rapid, easy detection and simultaneous testing for mutation frequency and drug resistance.

**Methods:**

In this study, the rifampicin and isoniazid resistance-related gene mutation chip method was employed for an epidemiological investigation. To assess the gene mutation characteristics of drug-resistant TB and evaluate the chip method, we tested 2143 clinical specimens from patients from the infectious diseases hospital of Changchun city from January to December 2016. The drug sensitivity test method was used as the reference standard.

**Results:**

The following mutation frequencies of sites in the rifampicin resistance gene *rpoB* were found: Ser531Leu (52.6%), His526Tyr (12.3%), and Leu511Pro (8.8%). The multidrug-resistance (MDR)-TB mutation frequency was 34.7% for *rpoB* Ser531Leu and *katG* Ser315Thr, 26.4% for *rpoB* Ser531Leu and *inhA* promoter − 15 (C → T), and 10.7% for *rpoB* His526Tyr and *katG* Ser315Thr. In addition, drug susceptibility testing served as a reference standard. In previously treated clinical cases, the sensitivity and specificity of GeneChip were 83.1 and 98.7% for rifampicin resistance, 79.9 and 99.6% for isoniazid resistance, and 74.1 and 99.8% for MDR-TB.

**Conclusions:**

Our experimental results show that the chip method is accurate and reliable; it can be used to detect the type of drug-resistant gene mutation in clinical specimens. Moreover, this study can be used as a reference for future research on TB resistance baselines.

**Electronic supplementary material:**

The online version of this article (10.1186/s12879-018-3131-8) contains supplementary material, which is available to authorized users.

## Background

According to a survey from the World Health Organization (WHO) in 2015, an estimated 1.4 million people have died from tuberculosis (TB) [1]. Although TB deaths decreased by 22% from 2000 to 2015, TB remained one of the top ten causes of death worldwide in 2015. In some areas, the proportion of multidrug-resistant (MDR)-TB in patients increased to a quarter of the TB cases on record. The WHO called for investing more funds for the treatment of MDR-TB to prevent the global spread of the disease. However, compared with conventional TB treatment, MDR-TB treatment is approximately 50 times to 200 times more expensive. Isoniazid (INH) and rifampicin (RFP) are the two most common first-line anti-TB drugs, but their widespread application has exacerbated resistance [2–4].

**Fig. 1:**
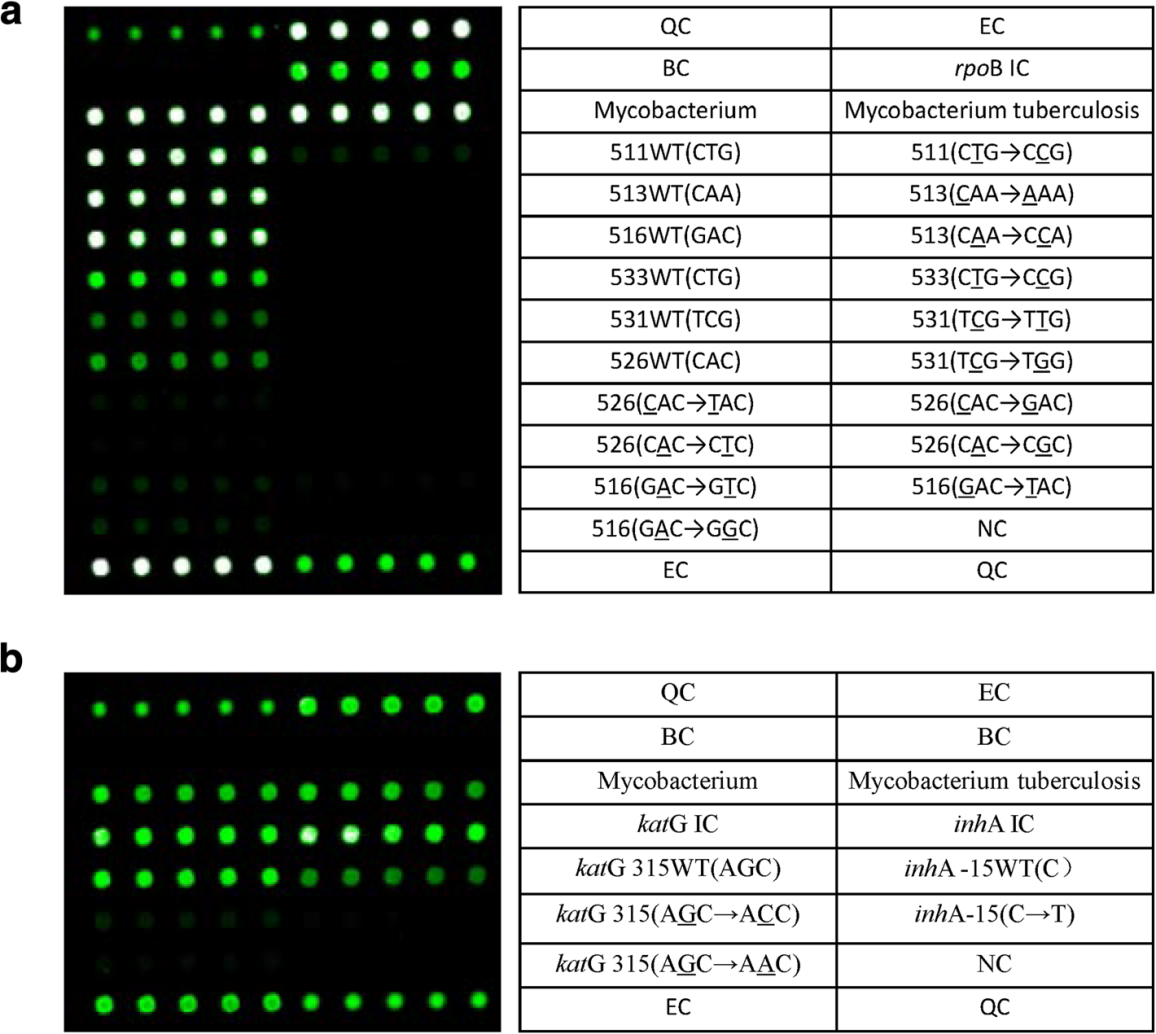
CapitalBio™ DNA microarray detection site layout. The contents of the table on the right side correspond to the microarray hybridization dot matrix on the left side in each figure. Every five repeated hybrid grid points correspond to one cell of specific content. QC: surface chemical quality control probe; EC: external control probe for hybridization-based quantitation; BC: blank control; NC: negative control probe; IC: internal control probe for PCR; WT: wild-type. **a**: Six sites detected in the *rpoB* gene, Ser531Leu (TCG → TTG), Ser531Trp (TCG → TGG), His526Asp (CAC → GAC), His526Tyr (CAC → TAC), His526Leu (CAC → CTC), His526Arg (CAC → CGC), Leu511Pro (CTG → CCG), Gln513Leu (CAA → CCA), Gln513Lys (CAA → AAA), Asp516Val (GAC → GTC), Asp516Tyr (GAC → TAC), Asp516Gly (GAC → GGC) and Leu533Pro (CTG → CCG), for a total of 13 types of mutants. **b**: The *katG* gene and a locus of the *inhA* gene promoter were tested as isoniazid resistance-related genes. The contents of the table on the right side correspond to the microarray hybridization dot matrix on the left side in each figure. Two katG gene mutants, Ser315Thr (AGC → ACC) and Ser315Asn (AGC → AAC), and one *inhA* gene promoter mutant, − 15 (C → T) mutant, were identified

Because the traditional drug susceptibility testing (DST) method is time consuming and cumbersome, a TB drug sensitivity test with an improved Löwenstein-Jensen medium and BACTEC MGIT 960 liquid culture is currently being implemented. However, waiting three to 4 weeks for the results is required after obtaining isolates, even if the BACTEC MGIT 960 liquid culture method is quickly applied. Furthermore, after a susceptible culture is identified, an additional 9–13 days are needed to obtain results; thus, the requirements for rapid clinical diagnosis are not being met [5–7]. A number of molecular techniques have been successfully applied to *Mycobacterium tuberculosis* isolates, including real-time polymerase chain reaction (RT-PCR), line probe assays (LPAs) and oligonucleotide or DNA microarrays. Development of oligonucleotide or DNA microarrays has proven feasible and practical in *M. tuberculosis* research [8, 9]. The CapitalBio™ DNA microarray method, which incorporates specific nucleotides at given positions of the *rpoB*, *inhA* and *katG* genes, has been developed to detect *M. tuberculosis* isolates and MDR forms in sputum specimens, with notable sensitivity and specificity. A previous study reported an accuracy of 91.8% for predicting RFP susceptibility and 70.2% for predicting isoniazid (INH) susceptibility compared with those of phenotypic DST, with detection in only 6 h [10, 11]. Among those genes assessed by the CapitalBio™ microarray, the following mutation sites in 13 isolates have been found in the RFP resistance-related gene *rpoB*: Ser531Leu (TCG → TTG), Ser531Trp (TCG → TGG), His526Asp (CAC → GAC), His526Tyr (CAC → TAC), His526Leu (CAC → CTC), His526Arg (CAC → CGC), Leu511Pro (CTG → CCG), Gln513Leu (CAA → CCA), Gln513Lys (CAA → AAA), Asp516Val (GAC → GTC), Asp516Tyr (GAC → TAC), Asp516Gly (GAC → GGC) and Leu533Pro (CTG → CCG). In addition, two mutants, Ser315Thr (AGC → ACC) and Ser315Asn (AGC → AAC), have been detected in the *katG* gene, as well as the − 15 (C → T) mutation in the *inhA* gene promoter [12] (Fig. 1).

In 2007, a national survey of drug-resistant TB was carried out in China but covered only 10 of 31 provinces, and Jilin Province was not included [13]. The city of Changchun is the capital of Jilin Province, and as a representative city of northeast China, it has a dense population and a large population flow. As the number of patients with TB is rising each year, assessing MDR-TB has become the main task of the current TB prevention and control program in the region. However, the city of Changchun has not yet been included in the TB drug-resistance statistics. Managing outbreaks of TB and studying epidemiological characteristics and resistance for the prevention and control of drug-resistant TB is of great significance for previously treated tuberculosis. At the same time, given that the two first-line drugs, namely, INH and RFP, are associated with resistance, the incidence of resistant TB is increasing and comprises a large proportion of the total cases [14]. In this study, previously treated tuberculosis patients in Changchun Infectious Disease Hospital were analyzed from January to December 2016. We used the CapitalBio ™ DNA microarray method and the DST approach as the reference standard to assess these cases in Changchun for *rpoB* and *inhA* mutations. We examined the molecular characteristics of *katG* gene mutation and correlations with INH and RFP resistance mutations with many clinical samples. Our results have important guiding value for clinical diagnosis and evaluation of developing trends in TB resistance.

This report is the first to describe a microarray analysis of mutations in the *rpoB*, *katG* and *inhA* genes of *M. tuberculosis* in a large number of clinical isolates in northeast China.

## Methods

### Clinical specimens

**Fig. 2:**
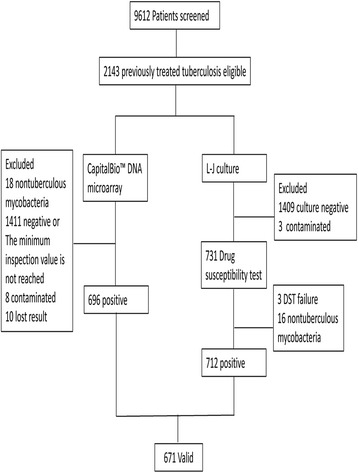
Study profile: Flow chart of tuberculosis subjects included in this study. Summary of the results of the two tests for 671 specimens

Patients with infectious diseases in the hospital of Changchun were included in this study. This hospital is the only designated tuberculosis hospital in Changchun; the number of outpatients was 71,139 from January to December 2016, and the number of hospitalized patients was 8890. Patients were assessed using the WHO Treatment of Tuberculosis: Guidelines [15]. This investigation was a retrospective study conducted from January 2016 to December 2016. Among the 9612 specimens screened, only those from patients with previously treated tuberculosis were included. In total, 2143 sputum samples were obtained for evaluation by DST and CapitalBio™ DNA microarray testing (Additional file 1). A total of 1409 cultures were negative, whereas 3 cultures were contaminated with other microbes. Sixteen cultures contained non-TB mycobacteria, and the DST results for 3 specimens were invalid. Of the specimens used for the CapitalBio™ DNA microarray test, 1411 were negative, or the amount of TB bacteria did not meet the minimum testing standards for *M. tuberculosis*. Eighteen specimens were identified as non-TB mycobacteria, 8 specimens were contaminated, and the results for 10 specimens were invalid. The results for 712 specimens were valid for DST evaluation. The results for 696 specimens were valid for GeneChip evaluation. In summary, the results of two tests revealed that 671 specimens were usable for evaluation of the performance of GeneChip. (Fig. 2).

### Culture and DST

DST was performed using Löwenstein-Jensen medium. The specimens were processed according to standard WHO procedures. The following critical concentrations were used: 0.2 μg/ml for INH and 40.0 μg/ml for RFP [16]. Staff members of all laboratories were trained and approved by the National Reference Laboratory of Tuberculosis.

### CapitalBio™ DNA microarray testing

**Fig. 3:**
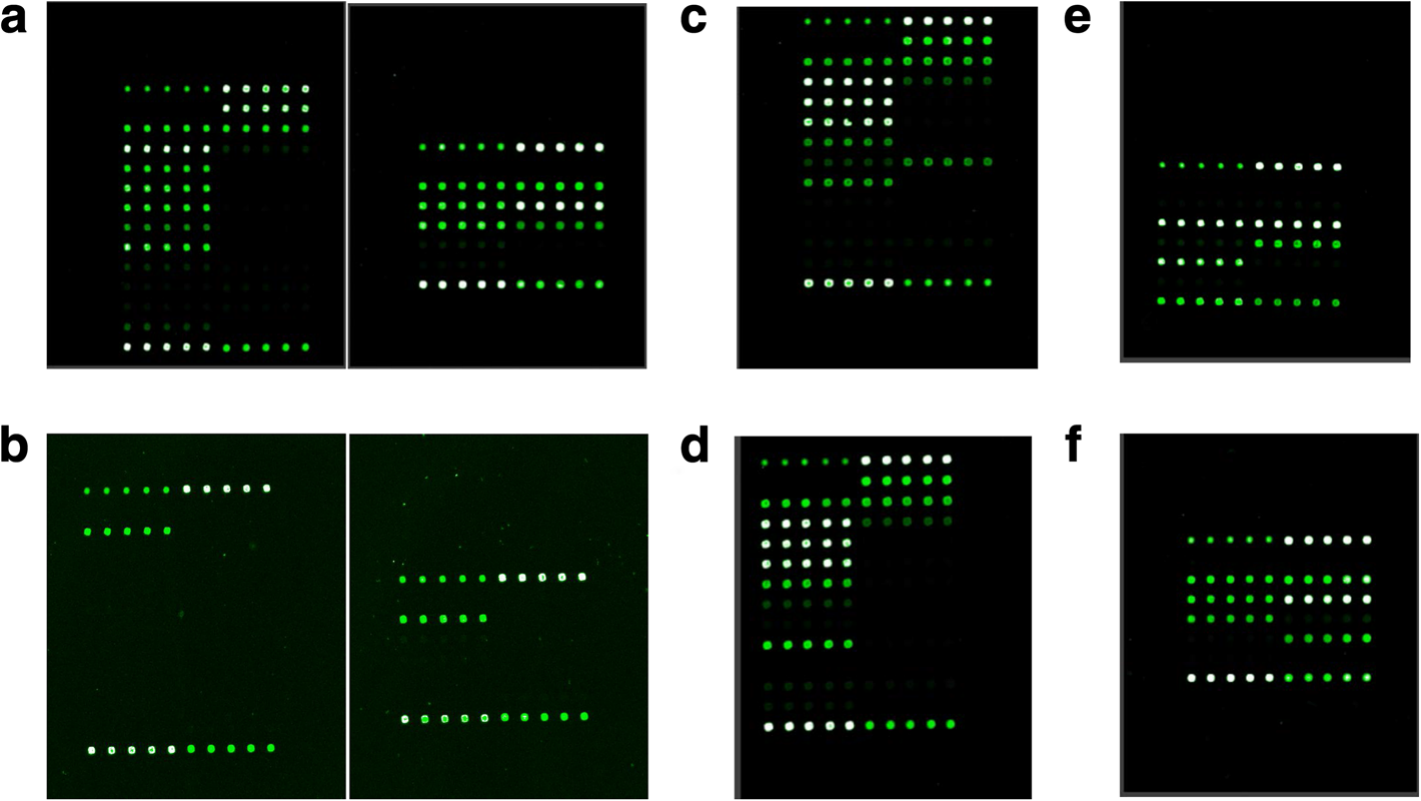
Common results of the CapitalBio™ DNA microarray detection spectra are shown for samples with mutation(s) at **a**: WT: wild-type. **b**: NTB: nontuberculous mycobacteria. **c**: *rpoB* gene codon 531 (TCG → TTG). **d**: *rpoB* gene codon 526 (CAC → TAC). **e**: *katG* gene codon 315 (AGC → ACC). **f**: *inhA* gene promoter − 15 (C → T)

For sputum sample collection and processing, the first sputum sample was collected in the early morning. After a clear water gargle, we first asked the patient to produce a deep, hard cough to raise sputum; the sample was deposited in sterile sample containers, sealed, and inspected. The samples were incubated 1 to 2 times in 4% NaOH, with agitation. After 15 to 20 min, we added mixed phosphate buffer, pH 6.8. After centrifugation, the supernatant was precipitated, and the pellet was washed with 0.5–1 ml of mixed phosphate buffer. The precipitates were then applied to the GeneChip according to the manufacturer’s instructions. The results were obtained via semi-automatic scanning using a LuxScan 10 K.B microarray scanner (CapitalBio Technology Inc., Beijing, China) (see Fig. 3 for common results).

### Statistical analyses

For data analysis, DST results were used as the reference standard to calculate the sensitivity, specificity, positive predictive value (PPV), and negative predictive value (NPV) of the CapitalBio™ DNA microarray. A chi-squared test was used for statistical analysis, and the criterion for significance was set at a *P* value of 0.05 based on a two-sided test. All statistics were performed with SPSS 17.0 software (Statistical Package for the Social Sciences, Inc., Chicago, IL, USA).

## Results

### CapitalBio™ DNA microarray test and DST results

The CapitalBio™ DNA microarray test was employed for 671 samples, including 437 wild-type samples, 57 with RFP resistance, 56 with INH resistance, and 121 with RFP and INH resistance. In addition, 671 samples were analyzed by DST, including 407 wild-type samples, 45 with RFP resistance, 57 with INH resistance, and 162 with RFP and INH resistance.

### Performance evaluation of the CapitalBio™ DNA microarray test for RFP and INH resistance among TB cases

The overall sensitivity, specificity, agreement rate, PPV, NPV, and kappa values were 83.1, 98.7, 93.9, 96.6, 92.9% and 0.85, respectively, for detection of *M. tuberculosis* RFP resistance. With regard to *M. tuberculosis* INH resistance, the overall sensitivity, specificity, agreement rate, PPV, NPV, and kappa values were 79.9, 99.6, 93.1, 98.8, 91.1% and 0.84, respectively (Table 1).

**Table 1:** Performance evaluation of the CapitalBio™ DNA microarray for rifampin and isoniazid resistance in tuberculosis cases compared with the standard drug sensitivity testing (DST) method for the 671 samples

CapitalBio™ DNA microarray	Conventional drug susceptibility testing								
	No.susceptible (%)	No.resistant (%)	Total No.	Sensitivity (%)	Specificity (%)	AR	PPV (%)	NPV (%)	Kappa
Rifampin				83.1	98.7	93.9	96.6	92.9	0.85
Susceptible	458 (98.7)	35 (16.9)	493						
Resistant	6(1.3)	172 (83.1)	178						
Total	464	207	671						
Isoniazid				79.9	99.6	93.1	98.8	91.1	0.84
Susceptible	450 (99.3)	44 (20.5)	494						
Resistant	2 (0.7)	175 (79.5)	177						
Total	452	219	671						

*Abbreviations*: *PPV* positive predictive value, *NPV* negative predictive value, *AR* agreement rate

For detecting *M. tuberculosis* MDR, the overall sensitivity, specificity, agreement rate, PPV, NPV, and kappa values were 74.1, 99.8, 93.6, 99.2, 92.4% and 0.81, respectively (Table 2).

**Table 2:** Performance evaluation of the CapitalBio™ DNA microarray for MDR-TB cases compared with the standard drug sensitivity testing (DST) method for the 671 samples

	Conventional drug susceptibility testing								
CapitalBio™ DNA microarray	MDR-TB (%)	non-MDR (%)	Total No.	Sensitivity (%)	Specificity (%)	AR	PPV (%)	NPV (%)	Kappa
				74.1	99.8	93.6	99.2	92.4	0.81
MDR-TB	120 (74.1)	1 (0.2)	121						
Non-MDR	42 (25.9)	508 (99.8)	550						
total	162	509	671						

*Abbreviations*: *PPV* positive predictive value, *NPV* negative predictive value, *AR* agreement rate

### Rifampicin resistance-related gene mutations in *rpoB*

Among the 57 cases with RFP resistance, the following mutations were found: 30 cases with Ser531Leu (52.6%), 7 cases with His526Tyr (12.3%), 2 cases with His526Leu (3.5%), 5 cases with Leu511Pro (8.8%), 1 case with Gln513Lys (1.8%), 2 cases with Asp516Val (3.5%), 3 cases with Asp516Tyr (5.3%), 2 cases with Asp516Gly (3.5%), 3 cases with Leu533Pro (5.3%), 1 case with Asp516TyrGln513LeuLeu511Pro (1.8%), and 1 case with Ser531LeuLeu511Pro (1.8%) (Table 3).

**Table 3:** Microarray chip detection of mutations in Mycobacterium tuberculosis rpoB-RRDR relevant mutation sites for the 57 samples

Codon mutation	Nucleic acid change	No. of strains	Frequency (%)
Ser531Leu	TCG → TTG	30	52.6
His526Tyr	CAC → TAC	7	12.3
His526Leu	CAC → CTC	2	3.5
Leu511Pro	CTG → CCG	5	8.8
Asp516Val	GAC → GTC	2	3.5
Asp516Tyr	GAC → TAC	3	5.3
Asp516Gly	GAC → GGC	2	3.5
Gln513Lys	CAA → AAA	1	1.8
Leu533Pro	CTG → CCG	3	5.3
Ser531LeuLeu511Pro	TCG → TTG CTG → CCG	1	1.8
Asp516TyrGln513LeuLeu511Pro	GAC → TAC CAA → CCA CTG → CCG	1	1.8
Total		57	

### Isoniazid resistance-related gene mutations in *katG* and *inhA*

Among the 56 cases with INH resistance, 30 cases had the *katG* 315 AGC → ACC mutation (53.6%), and 26 cases had the *inhA* -15 (C → T) mutation (46.4%) (Table 4).

**Table 4:** Mutations in katG and inhA gene in 56 rifampin-resistant M. tuberculosis isolates

Codon mutation	Nucleic acid change	No. of strains	Frequency (%)
katG			
Ser315Thr	AGC → ACC	30	53.6
inhA			
C(−15) → T	C → T	26	46.4
Total		56	

### Rifampicin and isoniazid resistance-related gene mutations in *rpoB*, *katG* and *inhA*

Among the 121 MDR-TB samples, 42 (34.7%) showed *rpoB* Ser531L and *katG* S315 T, 32 (26.4%) *rpoB* Ser531L and *inhA* promoter − 15 (C → T), and 13 (10.7%) *rpoB* His526Tyr and *katG* Ser315Thr (Table 5).

**Table 5:** Microarray chip detection of rpoB-RRDR,KatG315 and inhA-15 mutation points for the 121 samples

Codon mutation in rpoB	Codon mutation in katG	Codon mutation in inhA	No. of strains	Frequency (%)
Ser531Leu	Ser315Thr		42	34.7
Ser531Leu		C(−15) → T	32	26.4
Ser531Leu	Ser315Thr	C(−15) → T	2	1.7
Ser531LeuAsp516Tyr		C(−15) → T	2	1.7
Ser531LeuHis526Leu		C(−15) → T	1	0.8
His526Tyr	Ser315Thr		13	10.7
His526Tyr		C(−15) → T	4	3.3
His526Leu	Ser315Thr		2	1.7
His526Asp	Ser315Thr		2	1.7
His526AspGln513Lys	Ser315Thr		1	0.8
His526Arg	Ser315Thr		1	0.8
Asp516Val	Ser315Thr		2	1.7
Asp516Tyr	Ser315Thr		2	1.7
Asp516Gly	Ser315Thr		1	0.8
Asp516ValLeu511Pro		C(−15) → T	1	0.8
Asp516TyrLeu511Pro		C(−15) → T	1	0.8
Leu511Pro		C(−15) → T	2	1.7
Leu511Pro	Ser315Thr		2	1.7
Leu511ProAsp516Gly	Ser315Thr		2	1.7
Leu533Pro		C(−15) → T	2	1.7
Leu533Pro	Ser315Thr		2	1.7
Leu533Pro	Ser315Thr	C(−15) → T	1	0.8
Gln513Lys	Ser315Thr		1	0.8
Total			121	

## Discussion

### Evaluation of the DNA microarray method

TB infection is a serious health problem that threatens the health of people worldwide and creates a serious medical burden. TB detection methods primarily include sputum smear acid fast staining, DST methods and DNA microarray techniques. Popular in recent years, the DNA microarray method is a rapid detection approach. Indeed, chip methods are more rapid and accurate than culture and DST [17]. These techniques have the potential to guide the use of medication. Compared with culture and DST results, which are obtained in 6 weeks, DNA microarray results are obtained in 6 hours [11, 18]. In our study, we performed an evaluation using a large number of clinical samples and the DST method as a reference standard. High consistency was observed for the DNA microarray and DST methods. The overall sensitivity, specificity, agreement rate, PPV, NPV, and kappa values were 83.1, 98.7, 93.9, 96.6, 92.9% and 0.85 for detecting *M. tuberculosis* RFP resistance, respectively; 79.9, 99.6, 93.1, 98.8, 91.1% and 0.84 for detecting *M. tuberculosis* INH resistance, respectively; and 74.1, 99.8, 93.6, 99.2, 92.4% and 0.81 for detecting MDR-TB, respectively. These results were consistent with those reported by Guo, Y. et al., Pang, Y. et al., Tang, P et al., and Zhu, L. et al. [10, 19–21]. However, compared with the MeltPro TB assay method in detection of TB drug resistance [22, 23]. our chip method is less sensitive and has higher specificity.

### Limitations of the DNA microarray method

Based on the data shown in Table 1, RFP and INH mono-resistance positivity rates were 26.5% (178/671) and 26.4% (177/671), respectively, according to the DNA microarray method; these rates were slightly lower than the DST results at 30.8% (207/671) and 32.6% (219/671), respectively. The sensitivity values for RFP and INH were 83.1 and 79.9%, respectively, and the potential cause of this result is that some mutations occur beyond the limits of the chip testing sites. In some previous studies, the drug INH up-regulated the phenotypes of genes, such as *ahpC, kasA, NDH, iniABC, fadE*, and *furA*; thus, these gene phenotypes should be included in the detection range [2, 24, 25]. This phenomenon also led to further widening of the detection gap between the two methods; the positive rate for the chip method was 18.0% (121/671), whereas that of traditional culture DST was 24.1% (162/671). Increasing the number of detection sites in the DNA microarray test and expanding its detection range may help to increase sensitivity. In addition, some samples may have primary (natural) resistance [26, 27], as opposed to resistance caused by genetic mutations. For such cases, the CapitalBio™ DNA microarray method may need to be improved.

Although the chip method has limitations, it is still suitable for TB epidemic areas due to its rapid and accurate characteristics. Indeed, results can be quickly obtained with this tool compared with the traditional detection method, which requires 3 months or more for results. Thus, patients with MDR-TB can be quickly treated with an appropriate second-line treatment, with better outcomes.

### Drug-resistance gene mutations

*rpoB* gene mutation is the most important cause of RFP resistance. The main mutation in *rpoB* was the Ser531Leu mutation, which occurred in our samples, followed by mutations of residue 526. In similar previous reports, the 531 mutation was the most commonly detected, followed by the 526 mutation [28–32]. The results of our experiment revealed strains with mutations at three loci that were resistant to RFP (i.e., *rpoB* Asp516Tyr plus Gln513Leu plus Leu511Pro). Previously, this finding was rarely reported, and we should be aware of this possibility in the future.

Table 4 shows that INH mutations primarily occur at *katG* 315 and in the *inhA* promoter at − 15. For *katG* 315, AGC to ACC was the main mutation of this residue in our study, with 100% of cases showing the *katG* Ser315Thr AGC to ACC mutation, and we found no Ser315Asn AGC to AAC mutations, which was consistent with previous reports [21, 33]. However, in our study, *inhA* − 15 locus mutations accounted for 46.4% of all INH resistance mutations, which was inconsistent with previous reports [34–36]. Among the 56 cases of INH resistance mutations, 30 were *katG* 315 AGC → ACC (53.6%), and 26 were *inhA* -15 (C → T) (46.4%). Mutation rates of 14–17% and 22–24% have been reported for *katG* 315 AGC → ACC and *inhA* -15 (C → T), respectively [28, 34]; these values are very different from our results. This discrepancy may be caused by regional differences [2].

According to the results of our study, the resistance rates among the 671 *M. tuberculosis* strains with resistance were 26.5% (178/671), 26.4% (177/671) and 18.0% (121/671) for RFP mono-resistance, INH mono-resistance, and MDR, respectively. Our results were compared with a RFP resistance rate of 29.4%, an INH resistance rate of 38.5%, and a MDR rate of 25.6%, according to the results reported by Guo, Y. et al. [13]. However, the positive rates of RFP, INH and MDR resistance have varying degrees of decline; the reason for this reduction may be an increase in the use of second-line drugs. Although the use of these drugs would reduce the drug resistance of TB to first-line drugs, an increase in resistance rates to second-line drugs may lead to an increase in the overall prevalence of drug resistance [37]. A more scientific drug regimen and drug-resistant TB control should be further investigated.

## Conclusion

Changchun is a provincial capital city of northeast China, and the data reported herein are representative of this region. We conducted statistical analysis of sputum samples from hospitalized TB patients from January 2016 to December 2016 in the Changchun Infectious Disease Hospital. In recent years, there have been few reports of epidemiological investigations of TB resistance in China, and advances in detection techniques may lead to higher detection rates and more accurate results. Our results indicate that the DNA microarray method is a rapid, accurate, practical approach with promise for auxiliary clinical drug-resistant TB diagnosis. Investigating mutations in drug-resistance genes is important for the effective treatment of drug-resistant TB. To improve TB drug-resistance mutation detection, we should establish and improve an observation system and establish a new round of baseline investigation reports. Adopting the CapitalBio™ DNA microarray test to evaluate resistance will likely play a key role in this process.

## Additional file

Additional file 1: Raw data of 2143 simples. Clinical specimens test results from the infectious diseases hospital of Changchun city from January to December 2016. (XLSX 65 kb)
